# SECCA procedure for anal incontinence and antibiotic treatment: a case report of anal abscess

**DOI:** 10.1186/s12893-018-0389-0

**Published:** 2018-08-07

**Authors:** Francesca Mandolfino, Rosario Fornaro, Cesare Stabilini, Marco Casaccia, Tommaso Testa, Marco Frascio

**Affiliations:** 1 Dipartimento di Scienze Chirurgiche e Diagnostiche Integrate (DISC), Università degli Studi di Genova, Largo R. Benzi, 8, Genoa, Italy; 2 IRCCS Azienda Ospedaliera Universitaria Policlinico San Martino–IST, Largo R. Benzi, 8, Genoa, Italy

**Keywords:** SECCA procedure, Fecal incontinence, Anal abscess, Antibiotic treatment, Radiofrequency complication

## Abstract

**Background:**

Fecal Incontinence (FI) can seriously affect quality of life. The treatment of fecal incontinence starts conservatively but in case of failure, different surgical approaches may be proposed to the patient. Recently several not invasive approaches have been developed. One of these is the radiofrequency (RF) energy application to the internal anal sphincter.

**Case presentation:**

We report a rare case of an anal abscess related to a SECCA procedure in a 66-year-old woman affected by gas and FI for twenty years.

**Conclusions:**

The complications post-SECCA procedure reported in literature are generally not serious and often self-limited, such as bleeding or anal pain. This is a case of an anal abscess.

We suggest that this finding could consolidate the importance of administering antibiotic therapy to patients and to run a full course of at least 6 days rather than a short-term (24 h) therapy, with the aim to minimize the incidence of this complication.

## Background

Fecal incontinence (FI) is usually defined as the unintentional passing of stool in an inappropriate place or time more than two times a month. FI represents a worldwide problem affecting up to 12% of the general population and although is not considered a life-threatening disorder, it can seriously impair the quality of life of patients, frequently resulting in handicap [1, 2]. This disease is often under-diagnosed, under-reported and poorly managed. FI morbidity increases with age and is frequently observed up to 45% in over 70 years old population [3]. Treatment of FI is usually stratified from conservative to surgical treatments. The first approach starts with a fiber-enriched diet, physiotherapy of the pelvic floor and medication inducing constipation. When unsuccessful, a sphincter repair can be offered to patients presenting an anal sphincter defect. So far, the available surgical procedures include dynamic graciloplasty [4], sacral neuromodulation [5] and artificial bowel sphincter [6]. However, these treatments are often not completely satisfactory, carry considerable side effects and demand specific expertise. Moreover, data obtained in randomized trials are currently limited, and reliable guidelines for the optimal treatment of FI are still lacking. Current practice guidelines for FI treatment are mainly based on expert opinions, clinical experience, and case studies [7]. Recently, less invasive approaches for the treatment of FI have been developed. One of these is the application of RF energy to the internal anal sphincter, the SECCA procedure. For the last two decades RF energy has been already used to treat gastroesophageal reflux disease, prostatic hypertrophy, sleep apnea syndrome, ablation of hepatic tumors, spinal lesions, renal tumors, and joint capsule instability [8].

The SECCA procedure works on the principle that, once applied on the affected area, it induces modifications of the internal anal sphincter structure that should cause collagen deposition and fibrosis with the potential effect of tightening of the affected area. In particular, RF energy results in vibration of water molecules and subsequent frictional heating when delivered to a tissue in the frequency range of 200 kHz to 3.3 MHz [9–11]. Application of SECCA procedure in an animal model appeared to induce morphological changes in the internal and external anal sphincters leading to an anatomical state reminiscent of normal sphincter structure [12]. Thus, the SECCA procedure provides the release of temperature/impedance controlled RF energy to the sphincteric complex of the anal canal. In particular, the application of RF involves the anal canal up to 2.5 cm from the dentate line. The SECCA handpiece is an anoscopic device with four nickel-titanium curved needle electrodes (22 gauge, 6 mm in length) that are deployed through the mucosa of the anal canal into the internal anal sphincter muscle. The device is rotated four times of 90° in order to treat all the surface of the anal canal [8]. Major contraindications for the SECCA treatment are the anal Crohn disease [13, 14] and distal ulcerative colitis, as well as previous in loco radiotherapy (RXT). While bowel inflammatory diseases are contraindicated for local inflammatory status, RXT is contraindicated for the alterations induced in the tissue architecture of the anal canal.

## Case presentation

A 66 year-old woman presented with gas and FI for twenty years. She has one daughter, born in 1970 by cesarean section after a long labor without pelvic lesions or lacerations. She takes the following home therapy:

- Telmisartan 40 mg, 1 tablet at 12 h and 1 tablet at 20 h;
- Levotiroxine 75 mcg, 1 tablet at 8 h;
- Bromazepam 1,5 mg, 1 tablet at 8 h and 1 tablet at 20 h;
- Clomipramine 10 mg, 1 tablet at 8 h;
- Nebivol 5 mg, 1 tablet at 8 h.

She refers FI of liquid or solid stool and gas incontinence two-three times per day, which had a marked negative impact on her social life. She is suffering from anxiety-depressive syndrome that worsened because of incontinence. She has changed her lifestyle, her behavior and she is very embarrassed of her incontinence.

Physical examination: nothing to report.

Rectal exploration: anal sphincter hypotonia.

**Fig. 1:**
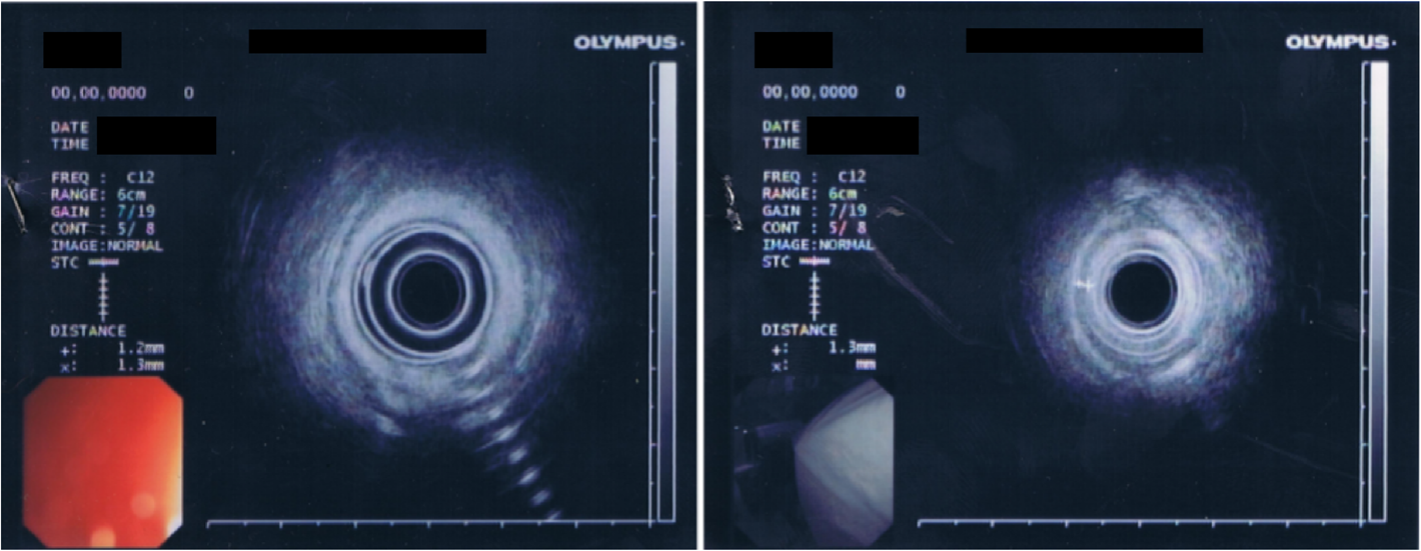
Transrectal endoscopic ultrasonography (TRUS). TRUS examination shows external anal sphincter echo structural normal. Internal anal sphincter without interruption of continuity, but of reduced thickness, about 1.3 mm measured at about 9 o’clock and 3 in correspondence of the middle part of the anal canal

Anorectal manometry was performed with detection of:

- Low median basal pressure: 20 mmHg (normal range 40–60 mmHg),
- The lower limit of normal pressure after maximal voluntary contraction: 93 mmHg,
- Duration of maximal voluntary contraction 15 s (normally more than 1 min),
- Sensitivity threshold to 30 ml (normally 40–60 ml),
- Threshold of subjective reflection to 40 ml (normal value 50–70 ml),
- Normal threshold of the inhibitory objective reflex: 40 ml (normal value 30–50 ml) (Fig. 1).

She did not perform any medical therapy for incontinence but she has performed 3 cycles of rehabilitation with anorectal biofeedback with poor benefit.

We proposed to the patient to undergo SECCA procedure.

Lithotomy position, general anesthesia was performed. A dose of 500 mg of metronidazole was administered intravenously to induction of anesthesia. Then, 20 applications of RF through the 4 nickel needles of the device were performed from the dentate line and proceeding cranially every 5 mm to 2.5 cm total. The same procedure was performed on the 4 quadrants of the internal anal sphincter, including the recto-vaginal wall (which is often the thinnest area and for this reason not always surgically treatable). The entire procedure lasted 40 min.

**Fig. 2:**
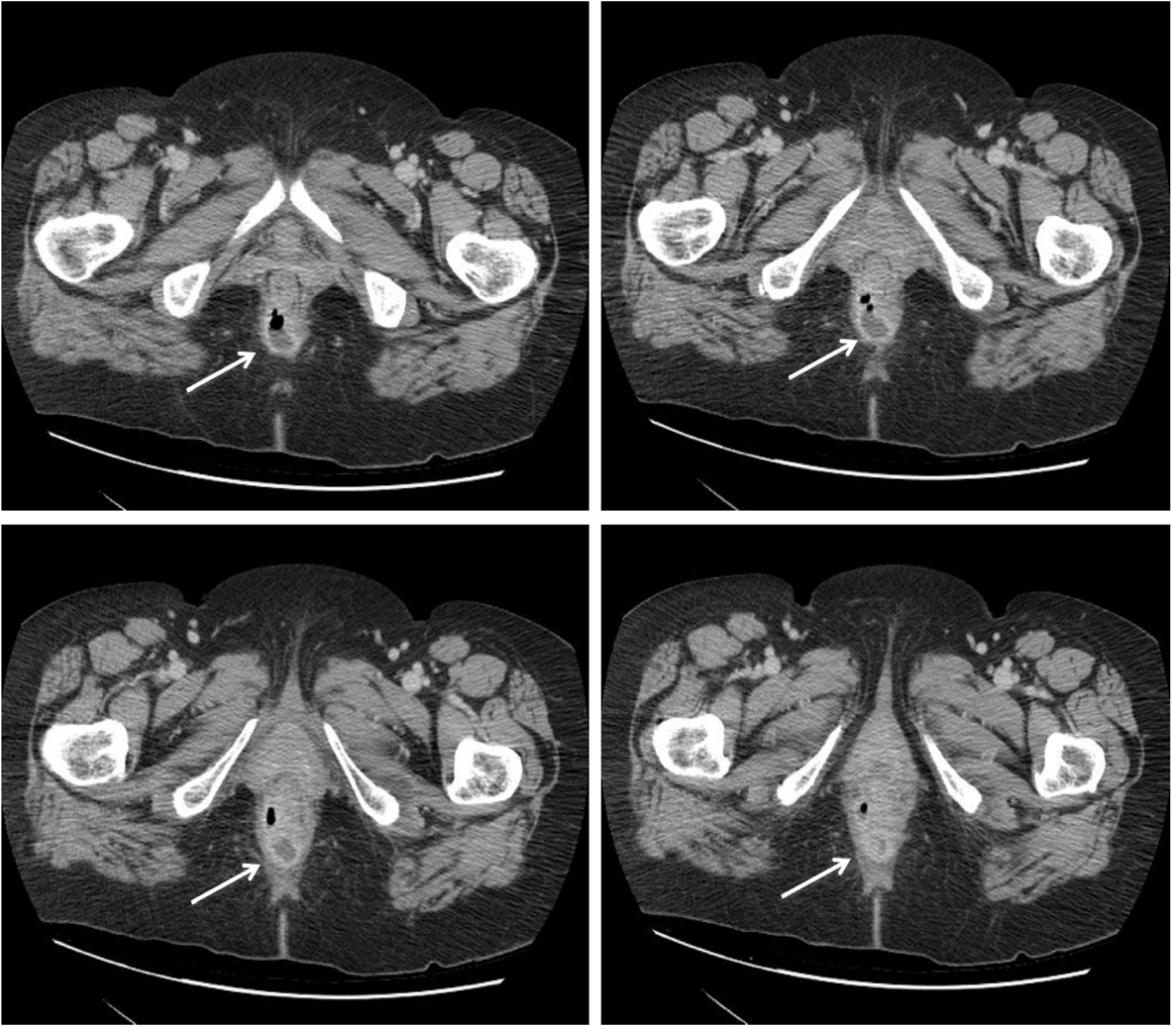
Abdominal CT scan. CT scan shows a small perianal lesion (32x30x28mm approximately) in the right posterior-lateral wall with hyperemic wall and partial gas content, probably an abscess (arrows). No free fluid in the pelvis

**Fig. 3:**
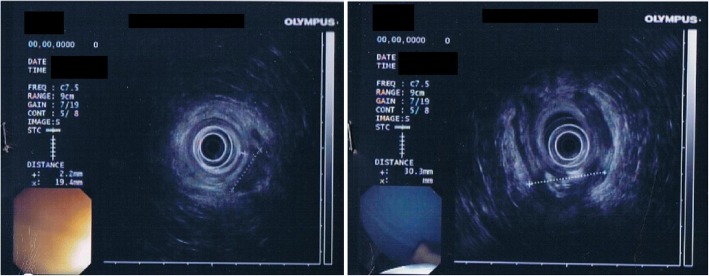
Transrectal endoscopic ultrasonography (TRUS). TRUS was performed to assess the extent of the abscess. TRUS shows normal endoscopic appearance of the rectal mucosa. Internal anal sphincter appears seamless continuity but with a thickness of about 2.2 mm. It was confirmed, in the right posterior-lateral area, the presence of an abscess 30 × 15 mm hypoechoic with hyperechoic images suggesting gas content

The day after surgery she was discharged in good health. After 10 days she presented intermittent hyperpyrexia, leak of purulent material through the anus and anal pain. We performed general physical and proctologic examination with anoscope and found evidence of abscess of the right posterior-lateral anal wall at 2 cm from the anal verge. We have sent the purulent material for bacterial culture and antibiogram: “*Escherichia coli* multi resistant”. The patient has performed blood tests without indices of inflammation replaying (Figs. 2 and 3). The authors administered antibiotic therapy with metronidazole and ciprofloxacin without satisfactory improvement of the symptoms.

Surgery has been organized to remove the abscess after 20 days from SECCA procedure.

**Fig. 4:**
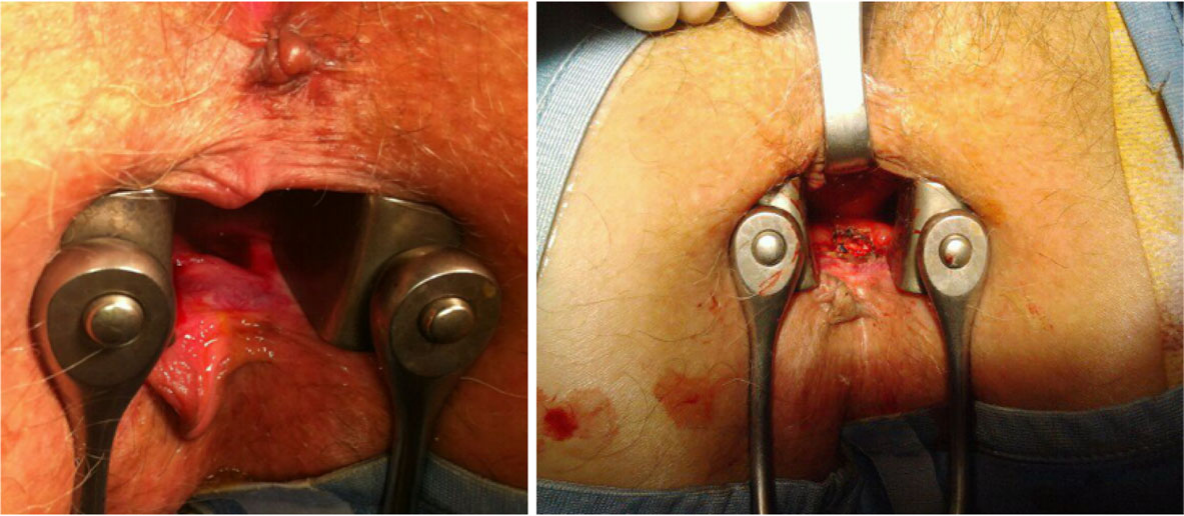
Surgical removal of the abscess. Opening and deroofing with curettage of the fundus treated the abscess

The patient was in lithotomy position. Metronidazole 500 mg was administered intravenously. The authors explored the anal canal finding about 2 cm from the anal verge, a recess of about 2–3 cm in diameter, undermined for about 1 cm in cranial direction. Opening and deroofing with curettage of the fundus treated the abscess (Fig. 4).

The day after surgery she was discharged in good health. Four days after the procedure, the patient was in good conditions. At 6 months follow up the patient was in good health and during the anal exploration it was possible to feel a rectal depression in the wall with a smooth consistence. In spite of the complication and subsequent surgical treatment, the procedure has been able to ameliorate the patient incontinence.

## Discussion and conclusion

Among the complications post-SECCA procedure, the finding of an anal abscess is rarely described in the literature [8]. Antibiotic therapy was not sufficient to treat the abscess but it was necessary a surgical treatment to cure this complication.

This case seems to consolidate the importance of administering antibiotic therapy to patients treated with SECCA procedure. We propose to run a full course (at least 6 days) rather than a short induction therapy, with the aim to minimize the incidence of complications.

## Abbreviations

CT: Computed tomography
FI: Fecal incontinence
RF: Radiofrequency
RXT: Radiotherapy
TRUS: Transrectal ultrasound
